# Diffusion-weighted MRI in solitary pulmonary lesions: associations between apparent diffusion coefficient and multiple histopathological parameters

**DOI:** 10.1038/s41598-018-29534-z

**Published:** 2018-07-26

**Authors:** Feng Zhang, Zien Zhou, Daoqiang Tang, Danni Zheng, Jiejun Cheng, Liaoyi Lin, Jianrong Xu, Xiaojing Zhao, Huawei Wu

**Affiliations:** 1 Department of Radiology, Ren Ji Hospital, School of Medicine, Shanghai Jiao Tong University, Shanghai, China; 2 The George Institute for Global Health, Faculty of Medicine, University of New South Wales, Sydney, Australia; 3 Department of Pathology, Ren Ji Hospital, School of Medicine, Shanghai Jiao Tong University, Shanghai, China; 4 Center for Big Data Research in Health, Faculty of Medicine, University of New South Wales, Sydney, Australia; 5 Department of Thoracic Surgery, Ren Ji Hospital, School of Medicine, Shanghai Jiao University, Shanghai, China

## Abstract

Apparent diffusion coefficient (ADC) from diffusion-weighted imaging (DWI) has gained wide attention as potential tool for differentiating between malignant and benign solitary pulmonary lesions (SPLs). The overall effects of multiple histopathological parameters on ADC have not been elucidated, which may help to explain the overlapping of ADC between malignant and benign SPLs. The study sought to explore associations between ADC and histopathological parameters in SPLs, and to compare diagnostic capability of ADC among different types of SPLs. Multiple histopathological parameters (cell density, nuclear-to-cytoplasm ratio, necrotic fraction, presence of mucus and grade of differentiation) were quantified in 52 malignant and 13 benign SPLs with surgical pathology available. Cell density (β = −0.40) and presence of mucus (β = 0.77) were independently correlated with ADC in malignant SPLs. The accurate diagnosis rate of squamous carcinomas, adenocarcinomas without mucus and malignant tumors with mucus was 100%, 82% and 0%, respectively. Our study suggested that cell density and presence of mucus are independently correlated with ADC in malignant SPLs. Squamous carcinoma maybe more accurately diagnosed as malignancy by ADC value. Malignant SPLs with mucus and adenocarcinomas with low cell density should be kept in mind in differentiating SPLs using ADC because of insufficient diagnostic capability.

## Introduction

Lung cancer is the leading cause of cancer-related death worldwide and is a global health burden^1^. Accurate and reliable diagnosis of pulmonary lesions using noninvasive imaging modality is needed in clinical practice. Prior studies have shown a beneficial effect of low-dose helical computed tomography (CT) in reducing mortality from lung cancer^2,3^. However, morphological assessment (size, shape, internal characteristics, lack of growth, etc.) of pulmonary lesion on CT is sometimes limited in differentiating malignant and benign lesions, especially for small lesions. Positron emission tomography (PET) using 18F-fluorodeoxyglucose (FDG) also has a contribution to differential diagnosis of pulmonary lesions^4,5^, but it is limited by false-negative results for well-differentiated pulmonary adenocarcinoma^6^, false-positive results for inflammatory nodules^7^, high cost and not widely available.

The use of chest magnetic resonance imaging (MRI) of solitary pulmonary lesion (SPL) is becoming increasing popular in clinical practice, as it obviates patient exposure to radiation and provides structural or functional information^8–10^. In particular, diffusion-weighted imaging (DWI) has gained wide attention as potential tool for differentiating between malignancy and benignity^11,12^, assessment of therapy response^13,14^, improving CT-guided transthoracic biopsy^15^ and evaluating N stage in patients with lung cancer^16^. DWI is sensitive to molecular diffusion and allows for microstructural characterization of tissue through probing changes of barriers to diffusion, which can be quantified as apparent diffusion coefficient (ADC)^17^. Although malignant pulmonary lesions have significantly lower ADC value than benign lesions in summary statistics^11^, ADC value may be not sufficiently robust to differentiate SPLs prospectively, because of diverse diagnostic thresholds reported in prior studies, confounding overlap with broad ranges, high heterogeneity in pooled sensitivity and specificity^12^.

Imaging-histopathological comparison study is helpful to identify the cause of overlapping ADC values between malignant and benign SPLs. Several histopathological parameters, such as cell density, nuclear-to-cytoplasm ratio (NCR), necrotic fraction and grade of differentiation, have been previously quantified and some were shown to be significantly correlated with ADC value in malignant SPLs^18–22^. However, to the best of our knowledge, the overall effects of these histopathological parameters on ADC value have not been elucidated. In this study, we aim to: (1) explore associations between ADC value and multiple histopathological parameters in SPLs, and (2) compare the diagnostic capability of ADC value and independently histopathological parameters among different types of SPLs.

## Results

### Included SPLs and pathological findings

From June 2014 to October 2016, 108 participants were consecutively recruited and underwent chest MRI. Two participants without histopathological diagnosis and 13 participants with inadequate image quality (4 with severe magnetic susceptibility artifacts and 9 with severe motion artifacts) were excluded. Of the remaining 93 SPLs (from 93 participants), 71 (76%) were malignant and 22 (24%) were benign. Characteristics of included participants and SPLs are shown in Tables 1 and 2. No significant difference was found in median size between malignant and benign SPLs (28 mm vs. 26 mm, P = 0.28). The major pathological diagnosis of malignant SPLs are adenocarcinoma, adenosquamous carcinoma, squamous cell carcinoma, small cell carcinoma, and metastasis. The types of benign SPLs include chronic inflammation, granuloma, organized pneumonia, chondroid hamartoma, bronchopulmonary sequestration, sclerosing hemangioma, hyperplastic lymphoid tissue, native benign smooth muscle lesion, and undetermined.

**Table 1:** Characteristics of included participants.

Characteristics	Participants with malignant SPLs (N = 71)	Participants with benign SPLs (N = 22)
Age (yr)—median ± IQR	61 ± 12	50 ± 30
Gender—no. (%)		
Female	32 (45.0)	18 (81.8)
Symptom—no. (%)		
Asymptomatic	44 (62.0)	17 (77.3)
Cough with bloody sputum	7 (9.9)	—
Cough without bloody sputum	13 (18.3)	2 (9.1)
Pyrexia	1 (1.4)	2 (9.1)
Chest pain	4 (5.6)	1 (4.5)
Hemoptysis	1 (1.4)	—
Hoarseness	1 (1.4)	—
Diagnostic method—no. (%)		
Surgical resection	52 (73.2)	13 (59.1)
Percutaneous puncture biopsy	7 (9.9)	3 (13.6)
Bronchoscopy biopsy	3 (4.2)	—
Mediastinoscopic biopsy	3 (4.2)	—
Pleural or distant metastasis	6 (8.5)	—
Follow-up	—	6 (27.3)

**Table 2:** Characteristics of SPLs included for analysis.

Characteristics	Malignant SPLs (N = 71)	Benign SPLs (N = 22)
Size of SPLs (mm) —median ± IQR	28 ± 15	26 ± 24^*^
Location of SPLs—no. (%)		
Left or right upper lobe	41 (57.7)	9 (40.9)
Left or right lower lobe	28 (39.4)	10 (45.5)
Right middle lobe	2 (2.8)	3 (13.6)
Pathological diagnosis—no. (%)		
	AC: 41 (57.7)	chronic inflammation: 3 (13.6)
	ASqC: 3 (4.2)	granuloma: 4 (18.2)
	SqCC: 9 (12.7)	organized pneumonia: 3 (13.6)
	SCLC: 6 (8.5)	chondroid hamartoma: 2 (9.1)
	metastasis: 4 (5.6)	BPS: 1 (4.5)
	others^†^: 8 (11.3)	SH: 1 (4.5)
		hyperplastic lymphoid tissue: 1 (4.5)
		native benign smooth muscle lesion: 1 (4.5)
		undetermined^**‡**^: 6 (27.3)

Abbreviations: AC, adenocarcinoma; ASqC, adenosquamous carcinoma; BPS, bronchopulmonary sequestration; SCLC, small cell lung cancer; SH, sclerosing hemangioma; SPL, solitary pulmonary lesion; SqCC, squamous cell carcinoma.

^*^P > 0.05 (Mann-Whitney U test) in the comparison of size between malignant and benign SPLs.

^†^Included 2 lung carcinoid, 2 sarcoma carcinoma, 1 mucinous adenocarcinoma, 1 mucoepidermoid carcinoma and 2 highly suspected malignant SPLs with more and enlarged metastases in brain, liver or bone after follow-up for 6 months.

^**‡**^Follow-up CT examinations for at least 2 years with no growth.

### Results of receiver operating characteristic analysis for ADC value in distinguishing malignant and benign SPLs

**Figure 1:**
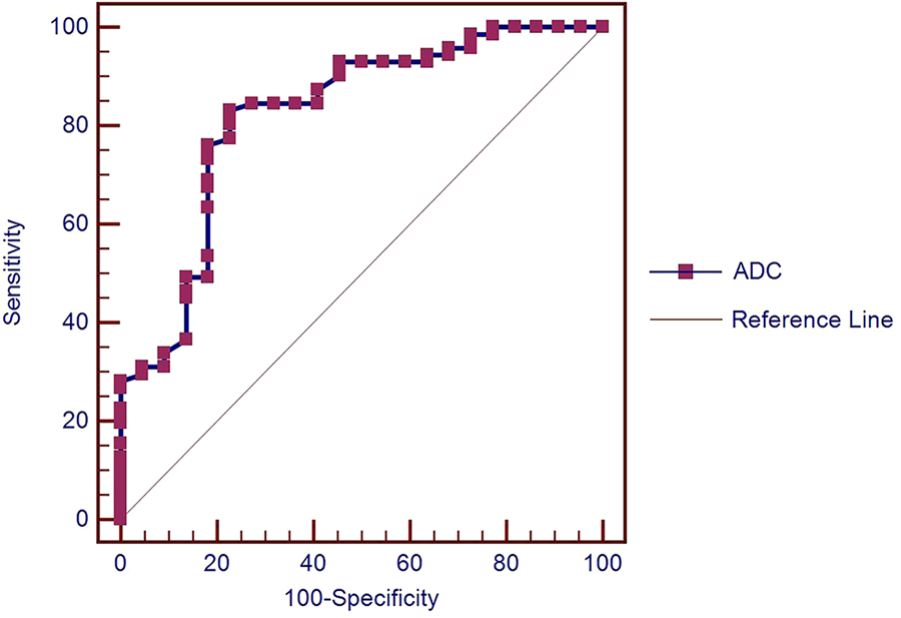
Receiver operating characteristic (ROC) curve for ADC value in distinguishing malignant and benign SPLs. The area under the ROC curve was 0.822 (95% confidence interval, 0.729 to 0.893).

The capability of ADC value in distinguishing malignant and benign SPLs were analyzed in the included 93 SPLs. Inter-reader agreement of ADC value was excellent with an ICC of 0.928 (95% confidence intervals [CI], 0.893 to 0.952). Mean ADC value in malignant SPLs was significantly lower than in benign SPLs (1.05 × 10^−3^ mm^2^/s vs. 1.49 × 10^−3^ mm^2^/s, P < 0.001). At optimal cut-off value of 1.21 × 10^−3^ mm^2^/s, ADC value could distinguish malignant and benign SPLs with sensitivity of 83.1% (95% CI, 72.3% to 91.0%), specificity of 77.3% (95% CI, 54.6% to 92.2%), PPV of 92.2% (95% CI, 82.7% to 97.4%) and NPV of 58.6% (95% CI, 38.9% to 76.5%) (see Fig. 1).

### Associations between ADC value and multiple histopathological parameters in malignant and benign SPLs

The associations between ADC value and histopathological parameters were analyzed in 52 malignant SPLs and 13 benign SPLs that were surgically excised. Cell density (β = −0.40, P < 0.001) and presence of mucus (β = 0.77, P < 0.001) were independently correlated with ADC value in malignant SPLs, whilst no significant associations were found in benign SPLs (see Table 3).

**Table 3:** Associations between ADC value and multiple histopathological parameters in malignant and benign SPLs.

Histopathological parameters	ADC in malignant SPLs (n = 52)	ADC in benign SPLs (n = 13)
	β^†^	β^†^
Cell density	−0.40^*^	−0.79
NCR	0.08	0.11
Necrotic fraction	−0.02	0.04
Presence of mucus	0.77^*^	NA
Grade of differentiation	0.05	NA
Adjusted R^2^	0.76	0.29
F (df)	34.07^*^(5, 46)	2.64 (3, 9)

Abbreviations: ADC, apparent diffusion coefficient; NA, not applicable; NCR, nuclear-to-cytoplasm ratio; SPL, solitary pulmonary lesion.

^*^P < 0.001.

^†^Standardized coefficient.

### Association between ADC value and single histopathological parameter in different types of SPLs, and diagnostic capability of ADC value and independently histopathological parameters in different types of SPLs

Of 52 malignant SPLs with surgical pathology available, 9 SPLs were squamous carcinomas, 31 SPLs were adenocarcinomas (27 without mucus, 3 with mucus production and 1 mucinous adenocarcinoma), 3 SPLs were adenosquamous carcinomas and 9 SPLs with other histopathological types (1 mucoepidermoid carcinoma, 1 small cell carcinoma, 2 lung carcinoid, 2 sarcoma carcinoma and 3 metastatic tumors). Correlation analysis between ADC value and single histopathological parameter, and type-specific accurate diagnosis rate were analyzed in 9 squamous carcinomas, 27 adenocarcinomas without mucus and 5 tumors with mucus (3 adenocarcinomas with mucus production, 1 mucinous adenocarcinoma and 1 mucoepidermoid carcinoma), respectively (see Tables 4 and 5).

**Table 4:** Association between ADC value and single histopathological parameter in different types of malignant SPLs^§^.

Spearman correlation coefficient	SqCC (n = 9)	AC(without mucus) (n = 27)	Tumor with mucus^†^ (n = 5)
r (ADC & cell density)	−0.85^*^	−0.57^*^	−0.80
r (ADC & NCR)	−0.50	0.20	−0.70
r (ADC & necrotic fraction)	0.22	—	—
r (ADC & GOD)	−0.64	0.04	0.35

Abbreviations: AC, adenocarcinoma; ADC, apparent diffusion coefficient; GOD, grade of differentiation; NCR, nuclear-to-cytoplasm ratio; SPL, solitary pulmonary lesion; SqCC, squamous cell carcinoma.

^§^Of 52 malignant SPLs with surgical pathology available, 11 lesions (3 adenosquamous carcinomas, 1 small cell carcinoma, 2 lung carcinoid, 2 sarcoma carcinoma and 3 metastatic tumors) were excluded for analysis because of small sample size.

^*^P < 0.01.

^†^Included 3 adenocarcinoma with mucus production, 1 mucinous adenocarcinoma and 1 mucoepidermoid carcinoma.

**Table 5:** Diagnostic capability of ADC value and independently histopathological parameters in different types of malignant SPLs^§^.

	SqCC^1^ (n = 9)	AC without mucus^2^ (n = 27)	Tumors with mucus^3*^ (n = 5)	P value (1 vs. 2)	P value (1 vs. 3)	P value (2 vs. 3)
ADR (%)	100%	82%	0%	0.21^†^	<0.001^†^	0.001^†^
ADC (×10^−3^ mm^2^/s)						
Mean ± SD	0.98 ± 0.11	1.08 ± 0.13	1.69 ± 0.18	0.04^**‡**^	<0.001^**‡**^	<0.001^**‡**^
Range	0.85–1.17	0.83–1.30	1.51–1.95			
Cell density						
Mean ± SD	373 ± 109	287 ± 51	275 ± 87	0.003^**‡**^	0.11^**‡**^	0.69^**‡**^
Range	253–610	209–391	205–399			

Abbreviations: AC, adenocarcinoma; ADC, apparent diffusion coefficient; ADR, accurate diagnosis rate; SPL, solitary pulmonary lesion; SqCC, squamous cell carcinoma.

^§^Of 52 malignant SPLs with surgical pathology available, 11 lesions (3 adenosquamous carcinomas, 1 small cell carcinoma, 2 lung carcinoid, 2 sarcoma carcinoma and 3 metastatic tumors) were excluded for analysis because of small sample size.

^*^Included 3 adenocarcinoma with mucus production, 1 mucinous adenocarcinoma, and 1 mucoepidermoid carcinoma.

^†^Chi-square test.

^**‡**^Student’s t-test.

**Figure 2:**
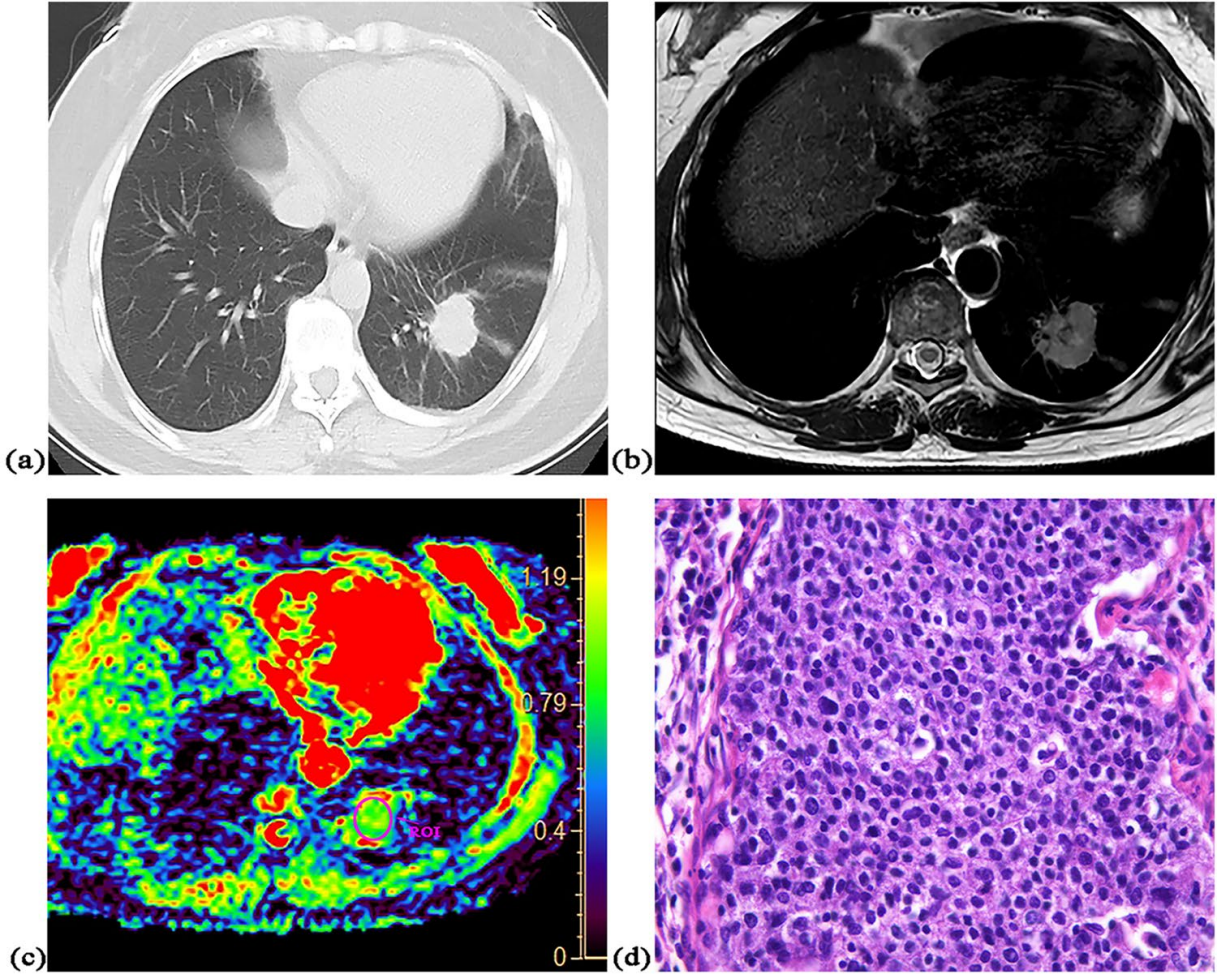
A 48-year-old female with squamous cell carcinoma at left lower lobe. (**a**) CT image, (**b**) T2-weighted image, (**c**) ADC map of DWI with placed ROI (mean ADC value in ROI is 0.86 × 10^−3^ mm^2^/s), (**d**) one field of view in histopathological slice for analysis (HE, magnification ×400, cell density: 610, nuclear-to-cytoplasm ratio: 0.68, necrotic fraction: class I, presence of mucus: no, high grade of differentiation).

**Figure 3:**
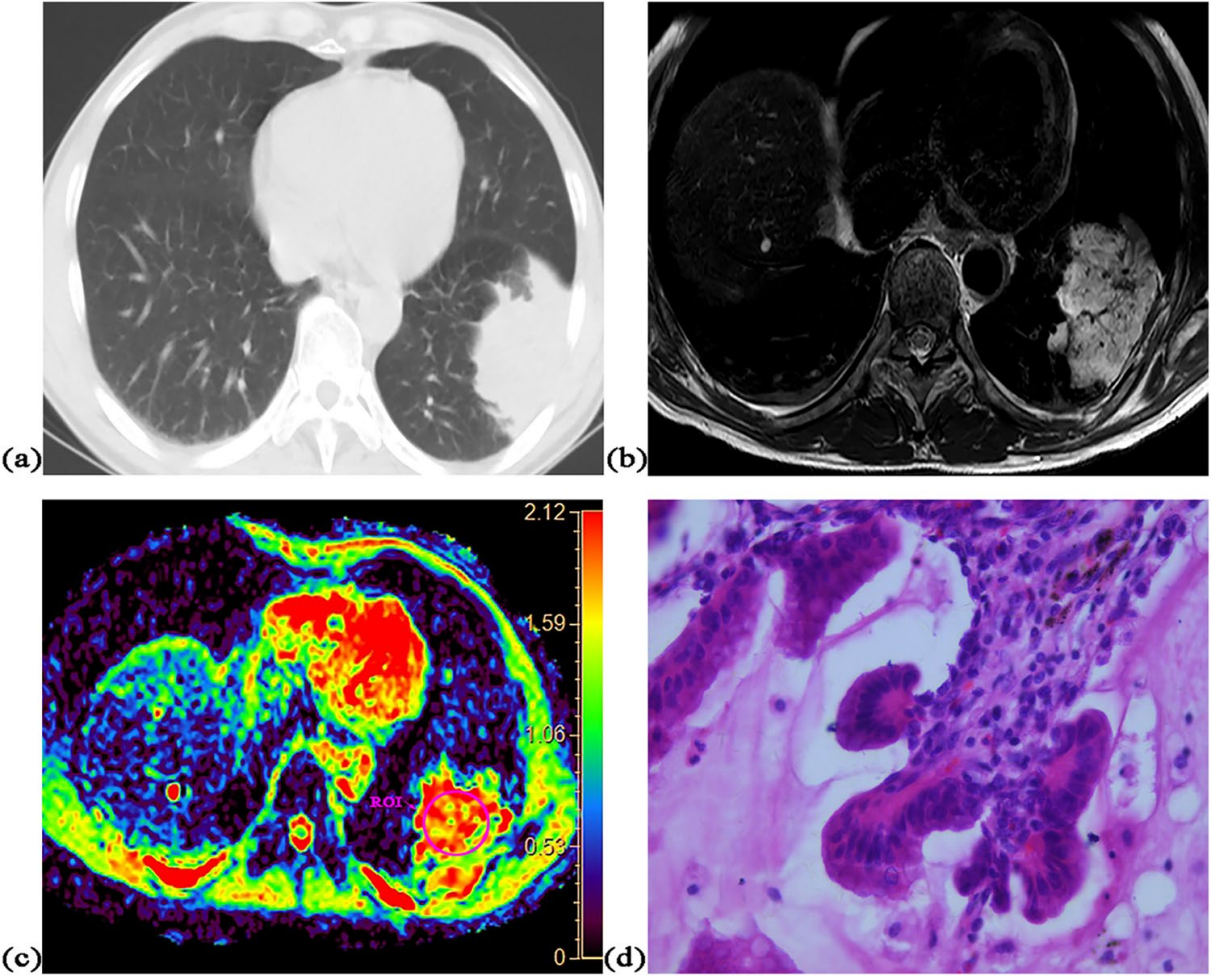
A 55-year-old male with mucinous adenocarcinoma at left lower lobe. (**a**) CT image, (**b**) T2-weighted image, (**c**) ADC map of DWI with placed ROI (mean ADC value in ROI is 1.95 × 10^−3^ mm^2^/s), (**d**) one field of view in histopathological slice for analysis (HE, magnification ×400, cell density: 205, nuclear-to-cytoplasm ratio: 0.25, necrotic fraction: class I, presence of mucus: yes, low grade of differentiation).

**Figure 4:**
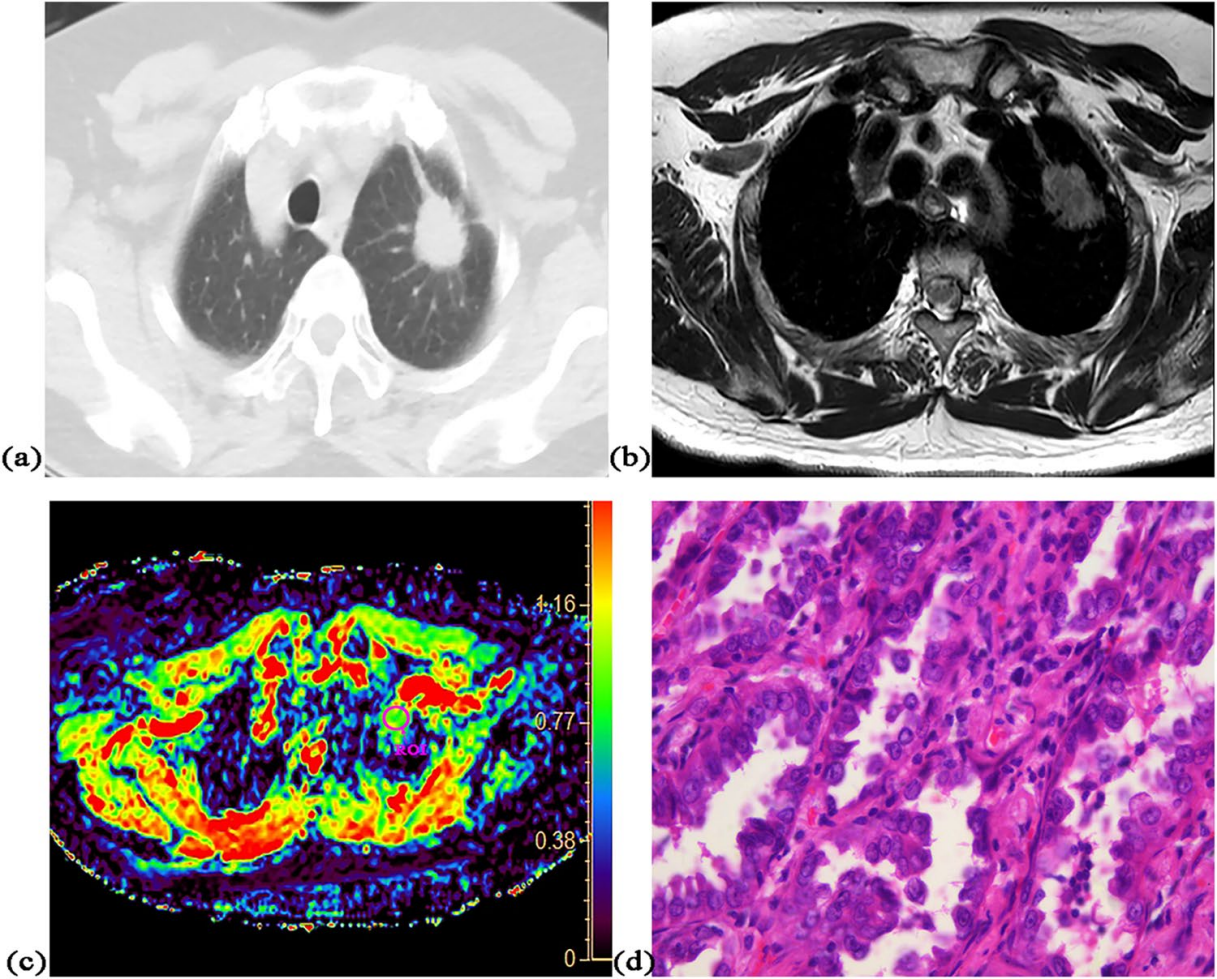
A 62-year-old female with adenocarcinoma at left upper lobe. (**a**) CT image, (**b**) T2-weighted image, (**c**) ADC map of DWI with placed ROI (mean ADC value in ROI is 1.01 × 10^−3^ mm^2^/s), (**d**) one field of view in histopathological slice for analysis (HE, magnification ×400, cell density: 279, nuclear-to-cytoplasm ratio: 0.33, necrotic fraction: class I, presence of mucus: no, low grade of differentiation).

There was significant correlation between ADC value and cell density in both squamous cell carcinoma (r = −0.85, P = 0.004) and adenocarcinoma without mucus production (r = −0.57, P = 0.002). The accurate diagnosis rate of squamous carcinomas was 100% (9/9), which was non-significantly higher than that [82% (22/27)] of adenocarcinomas without mucus (P = 0.21). Significant difference was found in mean ADC value (0.98 × 10^−3^ mm^2^/s vs. 1.08 × 10^−3^ mm^2^/s, P = 0.04) and mean cell density (373 vs. 287, P = 0.003) between them. All five malignant tumors with mucus could not be accurately diagnosed as malignancy according to optimal cut-off value. There was no significant difference in mean cell density between malignant tumors with mucus and adenocarcinoma without mucus (275 vs. 287, P = 0.69) or between malignant tumors with mucus and squamous carcinoma (275 vs. 373, P = 0.11). Figures 2–4 show representative cases of squamous carcinoma, mucinous adenocarcinoma and adenocarcinoma without mucus, respectively.

The ADC values and accurate diagnosis rates in different types of benign SPLs are shown in Table 6. There was significant difference in mean ADC value between inflammatory and noninflammatory benign SPLs (P = 0.001). The accurate diagnosis rate for noninflammatory benign SPLs was 100% (6/6). Because of the small sample size, we did not explore the association between ADC value and single histopathological parameter in benign subgroups.

**Table 6:** Diagnostic capability of ADC value in different types of benign SPLs.

Diagnosis	N	ADR (%)	ADC (×10^−3^ mm^2^/s)
Chronic inflammation^1^	3	67%	1.20 ± 0.25
Granuloma^1^	4	50%	1.16 ± 0.17
Organized pneumonia^1^	3	67%	1.30 ± 0.22
Chondroid hamartoma^2^	2	100%	2.14 ± 0.30
Bronchopulmonary sequestration^2^	1	100%	1.92
Sclerosing hemangioma^2^	1	100%	1.23
Hyperplastic lymphoid tissue^2^	1	100%	1.53
Native benign smooth muscle lesion^2^	1	100%	2.37
Undetermined^**‡**^	6	83%	1.53 ± 0.46
		P value (1 vs. 2): 0.074 ^*^	P value (1 vs. 2): 0.001^†^

Abbreviations: ADC, apparent diffusion coefficient; ADR, accurate diagnosis rate; SPL, solitary pulmonary lesion. ^*^Chi-square test. ^†^Student’s t-test.

^**‡**^Follow-up CT examinations for at least 2 years with no growth.

## Discussion

Associations between ADC value and multiple histopathological parameters in SPLs was analyzed in this imaging-histopathological comparison study. We found that cell density and presence of mucus were independently correlated with ADC value in malignant SPLs, but NCR, necrotic fraction and grade of differentiation were not. There was no significant association between ADC value and histopathological parameters in benign SPLs. ADC values from malignant SPLs with mucus or adenocarcinomas with low cell density were overlapping with ADC values from benign SPLs, which could induce false negative diagnosis.

Water diffusion in biological tissue is complicated and highly dependent on the ratio of extracellular to intracellular space, which can be reflected by cell density and NCR. Increasing cell density will decrease the extracellular space, and result in restriction of water diffusion in extracellular space. Increasing NCR may increase water diffusion in intracellular space, since intranuclear water has a higher diffusion coefficient than cytoplasmic water^23^. However, tumors with high NCR also tend to have a small amount of extracellular space. Chen *et al*.^21^ reported a negative correlation between NCR and ADC value in lung cancer. Our result indicated that confounding effect existed and water diffusion in intracellular space was likely less important in determining ADC value. In contrast, ADC value was independently affected by fraction of extracellular space as quantified by cell density. Apart from malignant SPLs, significantly negative correlation between ADC value and cell density was also found in other tumors^24–26^ and *in vitro* cultured cells^27^.

Animal studies^24,28–30^ in other tumors showed that presence of necrosis could reduce cell density and increase water diffusion. Usuda *et al*.^31^ reported contrary result in 63 patients with primary non-small cell lung cancer, however, such analysis is weak if necrosis is not independently correlated with water diffusion. In our study, only positive correlation was found in a small number of squamous carcinoma with no statistical significance. Lyng *et al*.^24^ suggested that necrotic regions should be comparable to or larger than voxel size of DWI so that partial volume effects could be avoided. In our histopathological analysis, necrotic regions with small size (mostly less than 2 × 2 mm size) was distributed diffusely in most (8 of 9) included squamous carcinomas, which may lead to non-significant result in correlation analysis.

Presence of mucus was another independently correlated parameter with water diffusion. To the best of our knowledge, only three studies reported the effect of mucus on ADC value in malignant SPLs^31–33^, and one study with more samples showed statistical significance^32^. Contrary to the result of several prior studies^18–20,22^, the present results failed to show correlation between grade of lung cancer and ADC value. Significant correlation between grade of differentiation and cell density as reported by Liu *et al*.^22^ might give some explanation to grade of differentiation as confounding parameter.

In terms of the diagnostic capability of ADC value in benign SPLs, we found that it was better in noninflammatory SPLs than in inflammatory SPLs which maybe caused by the overlap of ADC value between active inflammatory lesions and malignant lesions. Ciet *et al*.^34^ reported that ADC value of inflammatory lesion significantly increased after intravenous antibiotic treatment compared with baseline in patients with cystic fibrosis admitted for respiratory tract exacerbations treatment.

The repeatability of ADC measurement in our study was better than Bernardin *et al*.’s^35^ as we included more SPLs with size >2 cm or located at upper lobe. We noted that the proportion of excluded participants due to inadequate image quality for analysis was 12.0% (13/108), which was higher than that in prior studies by Yuan *et al*. (10.0%, 11/110)^10^ and Yan *et al*. (8.8%, 5/57)^14^. In our opinion, different DWI protocols may lead to potential difference in imaging quality of SPL. Respiratory-triggered gating technique was applied in our DWI sequence and part of prior studies^9,13,31,32^, while free breathing was adopted in others^8,10,14,21,22,35^. One recent study reported that ADC measurement for lung cancer had no significant difference in inter- and intraobserver agreement whatever free breathing, breath-holding or respiratory triggering was used^36^, but small samples (n = 22) and all included lesions were larger than 2 cm limit the statistical power of the result. Further high quality studies are still needed.

This study had some limitations. Firstly, the samples for benign SPLs and some types of malignant SPLs (squamous carcinoma, adenosquamous carcinoma and small cell lung carcinoma, etc) were relatively small. Since only 1 small cell lung carcinoma was surgically excised, comparison between small cell lung carcinoma and non-small cell lung carcinoma could not be performed. With only 9 participants with squamous carcinoma and 5 participants with tumors with mucus, our result on accurate diagnosis rate of ADC value in squamous carcinoma (with 100% accuracy) and tumors with mucus (with 0% accuracy) maybe too optimistic. Difference of diagnostic capability of ADC value among different types of malignant SPLs need to be further validated in studies with large sample size. Also, we did not make a precise correspondence between the site of histopathological analysis and that of ADC measurement. Point to point correspondence is necessary for radiologic-histopathologic comparison so that bias can be reduced, although it is technically difficult.

## Conclusions

Cell density and presence of mucus are independently correlated with ADC value in malignant SPLs. Squamous carcinoma maybe more accurately diagnosed as malignancy by ADC value because of higher cell density and lower ADC value. Malignant SPLs with mucus and adenocarcinomas with low cell density should be kept in mind in differentiating SPLs using ADC value because of insufficient diagnostic capability.

## Methods

### Participants

The prospective study was approved by the Ethic Committee of Shanghai Ren Ji Hospital, and the experimental protocols were performed in accordance with the approved guidelines. Informed consent was obtained from all of the participants before the study began. Patients with SPL (with size ≥ 1 cm, no air-containing area or calcification) found on CT and without resorption after 2 weeks of anti-infective treatment using oral or parenteral antibiotics were included. The exclusion criteria for participants were: (1) in poor physical condition, (2) with contraindications to MRI (heart pacemaker, metallic-implant or severe claustrophobia), (3) with subsolid pulmonary nodules, and (4) receiving percutaneous lung puncture biopsy, bronchoscopic biopsy or cancer-specific treatment before MRI examination.

### MR Imaging Protocol

Chest MRI examination was performed using a 3.0T whole-body full digital MR scanner (Ingenia, Philips Healthcare, Best, the Netherlands) with dStream Torso coil (an integrated body phased-array coil with up to 32 channels). Participants were in supine position throughout the scan. Anatomical MR imaging of SPLs was obtained by following sequences: (1) coronal T2-weighted turbo spin-echo (TSE): TR/TE 1250/80 msec, FOV 380 × 380 mm^2^, matrix 272 × 237, slice thickness/gap 4/0.5 mm, NSA = 1, single-shot; (2) axial T2-weighted TSE: TR/TE 1098/90 msec, FOV 250 × 250 mm^2^, matrix 280 × 245, slice thickness/gap 3/0.5 mm, NSA = 2, multi-shot; (3) axial.

T2-weighted spectral presaturation inversion recovery (SPIR) fat suppression: TR/TE 974/65 msec, FOV 250 × 250 mm^2^, matrix 252 × 210, slice thickness/gap 3/0.5 mm, NSA = 2, multi-shot; (4) axial mDixon TSE: TR/TE 3.5/1.2 msec, FOV 400 × 349 mm^2^, matrix 272 × 236, slice thickness/gap 1.75/0 mm, NSA = 1. Since the choice of b value for DWI in SPL has not come to an agreement and different b values were adopted in previous studies, DWI was performed using axial single-shot spin-echo echo-planar imaging (SE-EPI) sequence with multiple b-values (0, 250, 600, 800 and 1000 s/mm^2^) and following parameters: TR/TE 1044/69 msec, FOV 400 × 352 mm^2^, matrix 132 × 114, slice thickness/gap 3/0.3 mm, EPI factor 57, NSA = 1 for b value being 0 and 250 s/mm^2^, NSA = 2 for 600 and 800 s/mm^2^, NSA = 3 for 1000 s/mm^2^. SPIR fat suppression was used for DWI to reduce chemical shift artifact. Participants were requested to voluntarily suppress their breathing during the scan of mDixon TSE sequence. Respiratory-triggered gating technique was applied in other sequences. The total duration was approximately 15 minutes.

### Imaging Analysis

After scanning, ADC map of DWI was generated through mono-exponential fitting by total b values as derived from following equation: S_b_ = S_0_ exp(−b × ADC), where S_b_ is the signal intensity for a given b factor, and S_0_ is the signal intensity without diffusion weighting. The image quality of DWI/ADC maps was assessed by using three grades: grade 1, excellent, well-defined lesion contours, without distortion (due to magnetic susceptibility artifacts) and motion artifacts; grade 2, appropriate for analysis, identifiable lesion contours after referring to T2-weighted image, with moderate distortion or motion artifacts; grade 3, inadequate for diagnosis, obscured lesion contours, severe distortion or motion artifacts. SPLs with image quality of grade 3 were excluded from final analysis. A chest radiologist (J.C) with 15 years of experience in diagnosis of chest imaging, who was blinded to histopathological results, reviewed all images and measured ADC values. With reference to anatomical MR images, region of interest (ROI) was placed on the slice with largest SPL volume and encompassed entire lesion with a little distance from the edge. The mean ADC value in ROI was obtained for analysis. Lesion’s location and size (maximum diameter) on corresponding anatomical images were recorded. In order to assess the repeatability of ADC measurement, ADC values of included SPLs were measured again by another trained radiologist (L.L) with 3 years of experience in interpreting chest MRI.

### Histopathological Analysis

Surgically excised SPLs were analyzed by a pathologist (D.T) with 10 years of experience in diagnosis of pulmonary pathology. Cell density, NCR, necrotic fraction, presence of mucus and grade of differentiation were quantified as histopathological parameters in the slice with largest volume of specimen. A Leica DM5000 microscope (Leica Instruments Inc, Germany) coupled with a Leica digital sight DFC295 camera were applied for the conversion of histopathological slices to digital images.

Cell density was defined as an average cell count per five high-power fields, and NCR was estimated through dividing the percentage of nuclear area by that of cytoplasmic area^21,37^. For each lesion, five different fields of view (magnification ×400) were chosen randomly and digital images (with resolution of 2048 ×1536 pixels) were captured. ImageJ software package (National Institutes of Health, Bethesda, MD) was used for quantifying cell density and NCR in each field of view, and the average cell density and NCR in all fields of view were calculated. Necrotic fraction, presence of mucus and grade of differentiation were empirically estimated after reviewing the whole slice. Necrotic fraction was defined as the percentage of necrotic area in total lesion area^24^, and was classified into class I (<25%), class II (25% to 50%), class III (51% to 75%) or class IV (>75%). The grade of differentiation in malignant SPLs was categorized into low grade (well- and moderate-differentiation) and high grade (poor-differentiation) according to prior studies^18,19^.

### Statistical Analysis

Data were presented as mean ± standard deviation (SD), median ± interquartile range (IQR), range or count (percentage) as appropriate. Inter-reader agreement on ADC value was tested by one-way random intra-class correlation coefficient (ICC). Receiver operating characteristic (ROC) curve was used to calculate the sensitivity, specificity, positive predictive value (PPV) and negative predictive value (NPV) of ADC value in distinguishing between malignant and benign SPLs. In consideration of sample size, we included all SPLs with histopathological diagnosis (surgery or biopsy), distant organ metastasis or follow-up for at least two years without growth in analysis of ROC curve. For SPLs with surgical pathology available, associations between ADC value and multiple histopathological parameters were assessed using multiple linear regression in malignant and benign lesions, respectively. We also evaluated associations between ADC value and single histopathological parameters in different types of malignant SPLs (with sample size equal to or more than five) using Spearman correlation coefficient. On the base of optimal cut-off value according to Youden’s index from ROC curve, the type-specific accurate diagnosis rate was calculated retrospectively in different types of malignant and benign SPLs being surgically excised, which was defined as the percentage of malignant lesions with ADC value equal to or less than optimal cut-off value. Accurate diagnosis rate, ADC value and independently histopathological parameters among different types of malignant SPLs were compared by Chi-square test, student’s t-test or Mann-Whitney U test as appropriate. Normal or skewed distribution of continuous variables was assessed using Kolmogorov-Smirnov test. The SPSS software (version 22.0, Chicago, IL, USA) was used for statistical analysis. P < 0.05 was considered statistically significant.

### Data availability

The datasets generated and analysed during the current study are available from the corresponding authors on reasonable request.
